# Pressure-induced metallization in MoSe_2_ under different pressure conditions†

**DOI:** 10.1039/c8ra09441a

**Published:** 2019-02-15

**Authors:** Linfei Yang, Lidong Dai, Heping Li, Haiying Hu, Kaixiang Liu, Chang Pu, Meiling Hong, Pengfei Liu

**Affiliations:** Key Laboratory of High-Temperature and High-Pressure Study of the Earth's Interior, Institute of Geochemistry, Chinese Academy of Sciences Guiyang Guizhou 550081 China dailidong@vip.gyig.ac.cn; University of Chinese Academy of Sciences Beijing 100049 China; State Key Laboratory of Structural Chemistry, Fujian Institute of Research on the Structure of Matter, Chinese Academy of Sciences Fuzhou Fujian 350002 China

## Abstract

In this study, the vibrational and electrical transport properties of molybdenum diselenide were investigated under both non-hydrostatic and hydrostatic conditions up to ∼40.2 GPa using the diamond anvil cell in conjunction with Raman spectroscopy, electrical conductivity, high-resolution transmission electron microscopy, atomic force microscopy, and first-principles theoretical calculations. The results obtained indicated that the semiconductor-to-metal electronic phase transition of MoSe_2_ can be extrapolated by some characteristic parameters including abrupt changes in the full width at half maximum of Raman modes, electrical conductivity and calculated bandgap. Under the non-hydrostatic condition, metallization occurred at ∼26.1 GPa and it was irreversible. However, reversible metallization occurred at ∼29.4 GPa under the hydrostatic condition. In addition, the pressure-induced metallization reversibility of MoSe_2_ can be revealed by high-resolution transmission electron and atomic force microscopy of the recovered samples under different hydrostatic conditions. This discrepancy in the metallization phenomenon of MoSe_2_ in different hydrostatic environments was attributed to the mitigated interlayer van der Waals coupling and shear stress caused by the insertion of pressure medium into the layers.

## Abstract 2

Our experimental results clearly indicate that the metallization behavior of MoSe_2_ exhibits significant dependence on the pressure environments.

## Graphical abstract

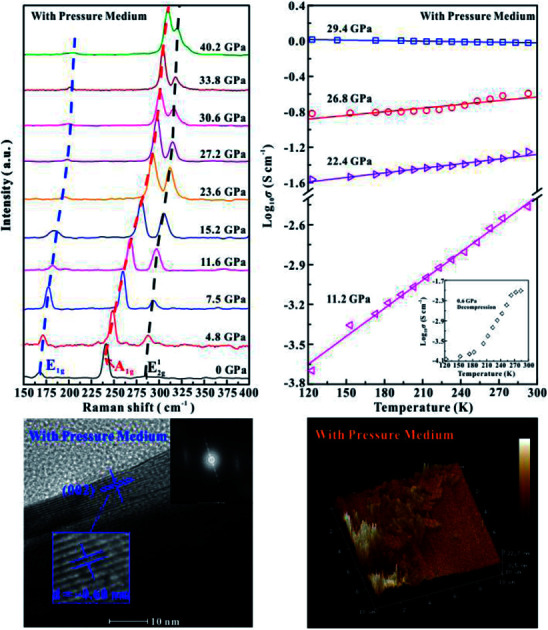

## Introduction

The AB_2_-type (A = Mo, W; B = S, Se, Te) transition-metal dichalcogenides (TMDs) have gained much interest owing to their unique mechanical, optical and electrical properties, and are widely used as solid-state lubricants, photodetectors, and transistors.^1–3^ Under ambient conditions, most of the transition-metal dichalcogenides possess a well-defined layered structure, in which the molecules are connected by the intralayer covalent bond and the interlayer van der Waals (vdW) forces. As an essential parameter, pressure can induce some changes in structural and electrical properties such as phase transition, amorphization and metallization in TMDs, and hence, pressure is of utmost importance for application in electromechanical devices, energy-variable opto-electronics and photovoltaics.^4–8^ Molybdenum diselenide (MoSe_2_) is one of the typical AB_2_-type TMDs with an indirect bandgap of ∼1.10 eV, crystallizing into a stable hexagonal 2*H*_c_ structure (space group: *P*6_3_/*mmc*) under ambient conditions.^9^ A systematic work on the high-pressure behaviour of MoSe_2_ can be helpful in understanding the crystalline structure evolution and electrical properties of AB_2_-type layered materials, and also promotes its industrial exploitation in electronic devices.

The high-pressure vibrational, structural and electrical properties for MoSe_2_ have been previously investigated by Raman and infrared spectroscopy, electrical resistivity and first-principles theoretical calculations. All of these results indicated that MoSe_2_ underwent a pressure-induced electronic transition from being a semiconductor to a metal. However, with regard to the metallization pressure point, there are many controversial viewpoints reported in previous studies. Rifliková *et al.* conducted the first-principles theoretical calculations up to 130 GPa to study the effect of high pressure on the evolution of the crystal structure and electronic properties of MoSe_2_, and predicted that it should undergo metallization at a pressure of at least ∼28 GPa.^10^ Caramazza *et al.* investigated the high-pressure optical properties of MoSe_2_ up to ∼30 GPa by means of Raman spectroscopy, and reported a pressure of ∼18 GPa for metallization with the pressure medium of NaCl.^11^ Dybala *et al.* measured the photoreflectance (PR) spectra of MoSe_2_ to explore its electronic band structure under hydrostatic pressure up to ∼20 GPa, and found that the metallization of MoSe_2_ occurred at ∼18 GPa with the pressure medium of liquid Daphne 7474.^12^ Zhao *et al.* investigated the high-pressure electrical transport behaviour of MoSe_2_ up to ∼60 GPa through the temperature-dependent electrical resistivity measurements, and their study results indicated that the metallization process was complete at ∼40 GPa using the pressure medium of cubic BN.^7^

In addition, as an important influential factor, the pressure environments can result in many structural and electronic property variations in the binary layered TMDs (*e.g.* WS_2_, MoS_2_ and ReS_2_). In the case of WS_2_, Duwal *et al.* confirmed that the isostructural phase transition from 2*H*_c_ to 2*H*_a_ occurred at ∼37 GPa under the non-hydrostatic condition, and however, under the hydrostatic condition, the phase structure was always stable with He being used as the pressure medium.^13^ Zhuang *et al.* observed a novel phenomenon that metallization of MoS_2_ was irreversible under the non-hydrostatic condition, while it was reversible under the hydrostatic condition.^14^ Zhuang *et al.* also found that metallization of ReS_2_ under the hydrostatic condition occurred at a much higher pressure point than that under the non-hydrostatic condition.^15^ As a similar layered structural diselenide, *viz.* MoSe_2_, it is possible that its high-pressure vibrational and electrical properties will be affected by different hydrostatic environments. However, till date, there are no relative reports on high-pressure structural and electronic properties in MoSe_2_ under different hydrostatic environments. Thus, further systematic investigation on MoSe_2_ is indispensable to explore the physical properties under different pressure environments.

In the present study, we report a semiconductor-to-metal transition of MoSe_2_ under non-hydrostatic and hydrostatic conditions of up to ∼40.2 GPa by virtue of a series of research methods including Raman spectroscopy, electrical conductivity, atomic force microscopy (AFM), high-resolution transmission electron microscopy (HRTEM) and first-principles calculations. Additionally, it was found that the metallization of MoSe_2_ was irreversible under the non-hydrostatic condition, but reversible under the hydrostatic condition. Furthermore, the reason for the diverse electrical properties displayed by MoSe_2_ under different pressure environments has been discussed in detail.

## Experimental

MoSe_2_ powder samples with a high purity of 99.99% were commercially purchased from Leshan Kaiyada limited company. The X-ray diffraction (XRD) analysis of the powders was performed using an X'Pert Pro X-ray powder diffractometer (Phillips; Cu Kα, 45 kV, 40 mA). The X-ray powder diffraction pattern of crystal MoSe_2_ is shown in Fig. S1 (ESI†) under ambient conditions. High-pressure Raman spectroscopy and electrical conductivity experiments were carried out using the diamond anvil cell (DAC) with two symmetric anvil culets of 300 μm. In this study, helium was used for all the hydrostatic measurements and no pressure-transmitting medium was adopted for the non-hydrostatic experiments. Pressure calibration was conducted by virtue of the wavenumber shift of ruby fluorescence peaks.^16^ The uncertainties of pressure calibration under non-hydrostatic and hydrostatic conditions were less than 5% and 3%, respectively. The Raman spectroscopic measurements at room temperature and high pressure conditions were performed using a Renishaw 2000 micro-confocal Raman spectrometer equipped with the 514.5 nm argon ion laser and the 20 mW laser power. Each Raman spectroscopy was gathered in the frequency shift range of 150–400 cm^−1^ with a spectral resolution of ∼1.0 cm^−1^, and the collection time for each spectrum was ∼120 s. As for electrical conductivity measurements at room temperature and high pressure conditions, a T-301 stainless steel was employed as the gasket and pre-indented to a thickness of 50 μm beforehand, and then a 180 μm hole was drilled in the centre of the gasket by the laser drilling technique. The mixture of boron nitride (BN) and epoxy was compressed into the hole as the insulator, and another hole of 100 μm was then drilled to provide an insulated sample chamber. The electrical conductivity measurements were carried out by two-electrode method using a Solartron-1260 impedance/gain phase analyzer in the frequency range from 10^−1^ to 10^7^ Hz. The temperature-dependent electrical conductivity measurements were performed in the temperature range from 120 K to 300 K. Similar to the experimental technique by Rahman *et al.*, the *in situ* high-pressure electrical resistivity experiment is valid up to 55 GPa in the diamond anvil cell.^17^ More detailed descriptions on the experimental procedures and measurement methods have been reported previously.^18–20^

Some microscopical observations on the recovered samples were performed at room temperature and high pressure conditions by virtue of the cross-sectional selected-area high-resolution transmission electron and atomic force microscopy. All of the acquired data *via* atomic force microscopy (AFM) and high-resolution transmission electron microscopy (HRTEM) images were collected using a Multimode 8 mass spectrometer (Bruker) and Tecnai G2 F20 S-TWIN TMP, respectively. The first-principles calculations were conducted on the basis of the density functional theory and pseudopotential methods, which were performed using the CASTEP code in the Material Studio package. The generalized gradient approximations (GGA) in the Perdew–Burke–Ernzerhof (PBE) scheme were applied for the description of the exchange and correlation terms. Structural optimizations were implemented using the Broyden–Fletcher–Goldfarb–Shanno (BFGS) minimization algorithm. In order to ensure high convergence of the enthalpy calculations, the cutoff energy and *K*-point grid were set to 950 eV and 24 × 24 × 8, respectively.

## Results and discussion

**Fig. 1:**
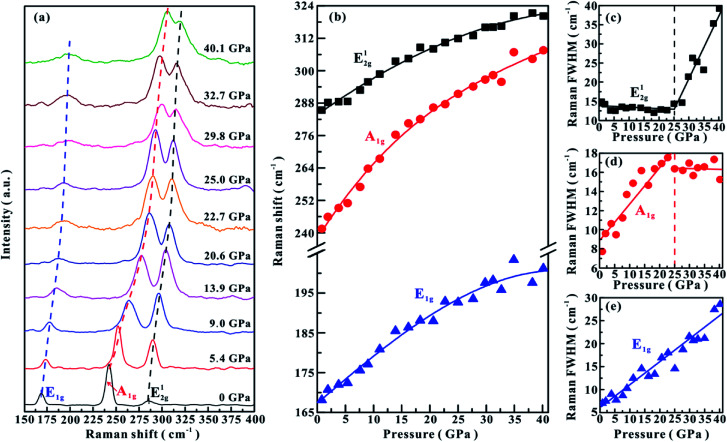
(a) Raman spectra of MoSe_2_ at representative pressure points under the non-hydrostatic condition. (b) The Raman shifts of three vibrational modes with increasing pressure. (c)–(e) The pressure dependence of FWHM for E^1^_2g_, A_1g_ and E_1g_, respectively. The dashed and solid lines serve as visual guides.

**Fig. 2:**
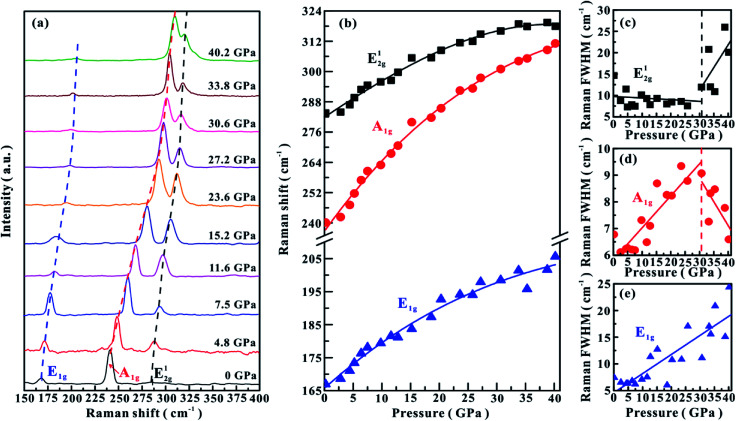
(a) Raman spectroscopic results of MoSe_2_ at representative pressure points under the hydrostatic condition. (b) The Raman shifts of three vibrational modes with increasing pressure. (c)–(e) The pressure dependence of FWHM for E^1^_2g_, A_1g_ and E_1g_, respectively. The dashed and solid lines serve as visual guides.

The high-pressure vibrational properties of MoSe_2_ were explored using Raman spectroscopy up to ∼40.2 GPa at room temperature. Raman spectra of MoSe_2_ were measured under both non-hydrostatic and hydrostatic conditions, and the corresponding results are displayed in Fig. 1a and 2a, respectively. Under ambient conditions, three typical Raman-active modes in MoSe_2_ were observed: A_1g_ (241 cm^−1^), E_1g_ (168 cm^−1^) and E^1^_2g_ (285 cm^−1^), which are in good agreement with previously reported results.^21,22^ A_1g_ is an out-of-plane vibrational mode associated with the weak interlayer vdW interactions, while E_1g_ and E^1^_2g_ are in-plane vibrational modes related to the strong intralayer covalent bonds.^12^

Under the non-hydrostatic condition, as shown in Fig. 1b, all of these obtained modes monotonously shift to higher wave numbers with an increase in the pressure up to ∼40.1 GPa, showing no anomaly in the pressure-dependent Raman shifts. More specifically, it can be seen that these vibrational modes shift at different rates: the A_1g_ mode shifts at much larger rate than E_1g_ and E^1^_2g_ modes. However, according to the variations in the full-width at half-maximum (FWHM) for E^1^_2g_ and A_1g_ modes, one distinct inflection point was obtained at a pressure of ∼25.0 GPa. The FWHM of E^1^_2g_ mode gradually decreases in the pressure range of 0–25.0 GPa, but increases with pressure in the range of 25.0–40.1 GPa (Fig. 1c). The FWHM of A_1g_ mode displays different pressure dependence: it increases with pressure below ∼25.0 GPa, but decreases with pressure above ∼25.0 GPa (Fig. 1d). The FWHM of E_1g_ mode continuously shifts to higher wave numbers with pressure (Fig. 1e). Under the hydrostatic condition, the pressure-dependent Raman shifts of three modes show monotonously increasing trends, and no anomalous pressure point was observed up to ∼40.2 GPa (Fig. 2b). As for the FWHM of E^1^_2g_ and A_1g_ modes, we obtained a discontinuous point at a higher pressure of ∼30.6 GPa than those observed under the non-hydrostatic condition (Fig. 2c and d). The E_1g_ mode exhibits a monotonously increasing tendency with pressure in the FWHM (Fig. 2e).

In terms of these observed discontinuities in the FWHM of E^1^_2g_ and A_1g_ modes, Raman spectroscopy is one efficient method in determining the metallization of MoSe_2_ since the FWHM of these modes are directly associated with the charge transfer processes, which has been widely used to probe the metallization process in other materials.^6,23^ However, the metallization pressures under non-hydrostatic and hydrostatic conditions are determined to be ∼25.0 and ∼30.6 GPa, respectively. The discrepancy in the metallization pressure points was possibly related to the deviatoric stress. Under the non-hydrostatic condition, there exists strong deviatoric stress in the sample chamber (as displayed in Fig. S2,† the deviatoric stress reaches ∼5 GPa when the centre pressure is ∼39.6 GPa), which facilitates the occurrence of metallization by accelerating the reduction of the interlayer distances. However, for the hydrostatic condition, this electronic transition is delayed due to the protection by the pressure medium, which enters into the interlayer space and thus weakens the deviatoric stress.

**Fig. 3:**
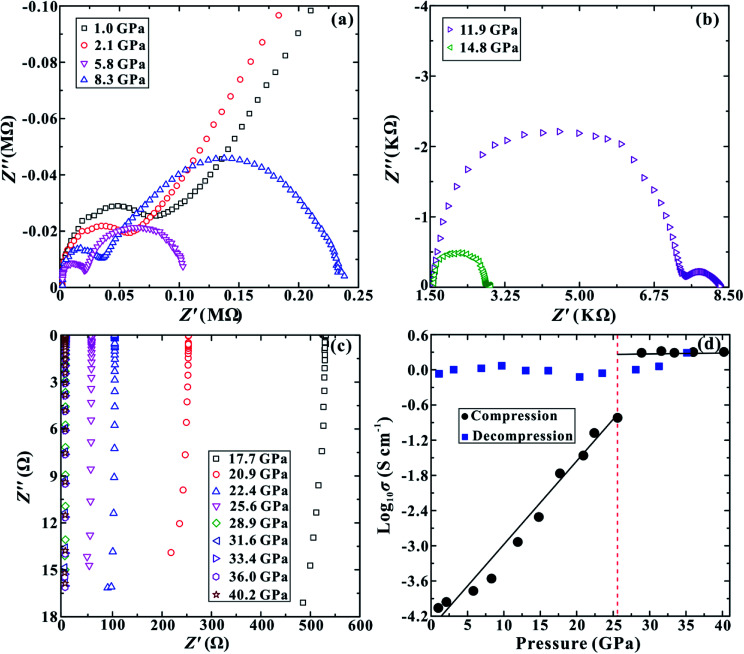
(a)–(c) The impedance spectra of MoSe_2_ in pressure ranges of 1.0–40.2 GPa. The horizontal and vertical axes represent the real and imaginary parts of the complex impedance, respectively. (d) The pressure dependence of electrical conductivity for MoSe_2_ in the process of compression and decompression.

The room-temperature and high-pressure electrical conductivity measurements have been implemented to explore the electrical behaviour of MoSe_2_ under the non-hydrostatic condition. The representative impedance spectra of MoSe_2_ up to ∼40.2 GPa are presented in Fig. 3a–c. In the pressure range of 1.0–14.8 GPa, the impedance spectra exhibit one approximately semicircular arc at high frequencies and another small semicircular arc at low frequencies, which stand for the grain interior and grain boundary contribution, respectively (Fig. 3a and b). Both semicircular arcs gradually decrease with the increase in the pressure from ∼1.0 to ∼14.8 GPa, and the grain boundary contribution of the sample becomes weaker. When the pressure is enhanced to ∼17.7 GPa, as shown in Fig. 3c, the grain boundary arc disappears and only the grain interior semicircular arc appears in the fourth quadrant, implying the occurrence of pressure-induced electronic polarization.^24^Fig. 3d shows the pressure-dependent electrical conductivity of MoSe_2_ in the process of compression and decompression. One evident discontinuous point is observed at a pressure of ∼25.6 GPa upon compression. The electrical conductivity of the sample continuously increases in the pressure range of 1.0–25.6 GPa, but it tends to be stable with a relatively high value of ∼2.0 S cm^−1^ in the pressure range of 28.9–40.2 GPa. This available discontinuity of the electrical conductivity provides a robust evidence for the metallization of MoSe_2_ at ∼25.6 GPa, and this pressure point is consistent with the above-mentioned FWHM results under the non-hydrostatic condition. Upon decompression, the electrical conductivity of MoSe_2_ still remains at a very high value of ∼1.0 S cm^−1^, providing a crucial clue that the metallization is irreversible. This pressure-induced irreversible metallization is a very novel phenomenon, which has also been confirmed to occur in some other layered materials, such as MoS_2_, α-As_2_Te_3_ and Sb_2_S_3_.^14,18,19^

**Fig. 4:**
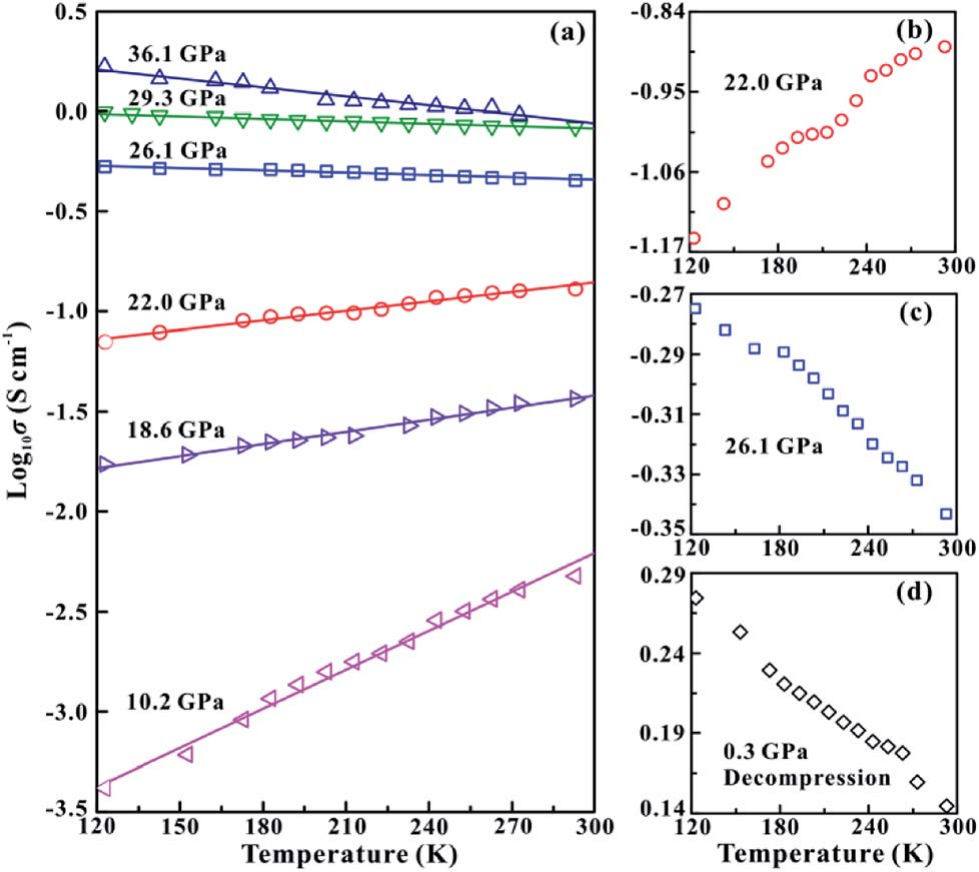
(a) The temperature-dependent electrical conductivity of MoSe_2_ at different pressure points under the non-hydrostatic condition. (b) The semiconducting state of MoSe_2_ at ∼22.0 GPa. (c) The metallization of MoSe_2_ at ∼26.1 GPa. (d) The metallic state of MoSe_2_ after decompression.

**Fig. 5:**
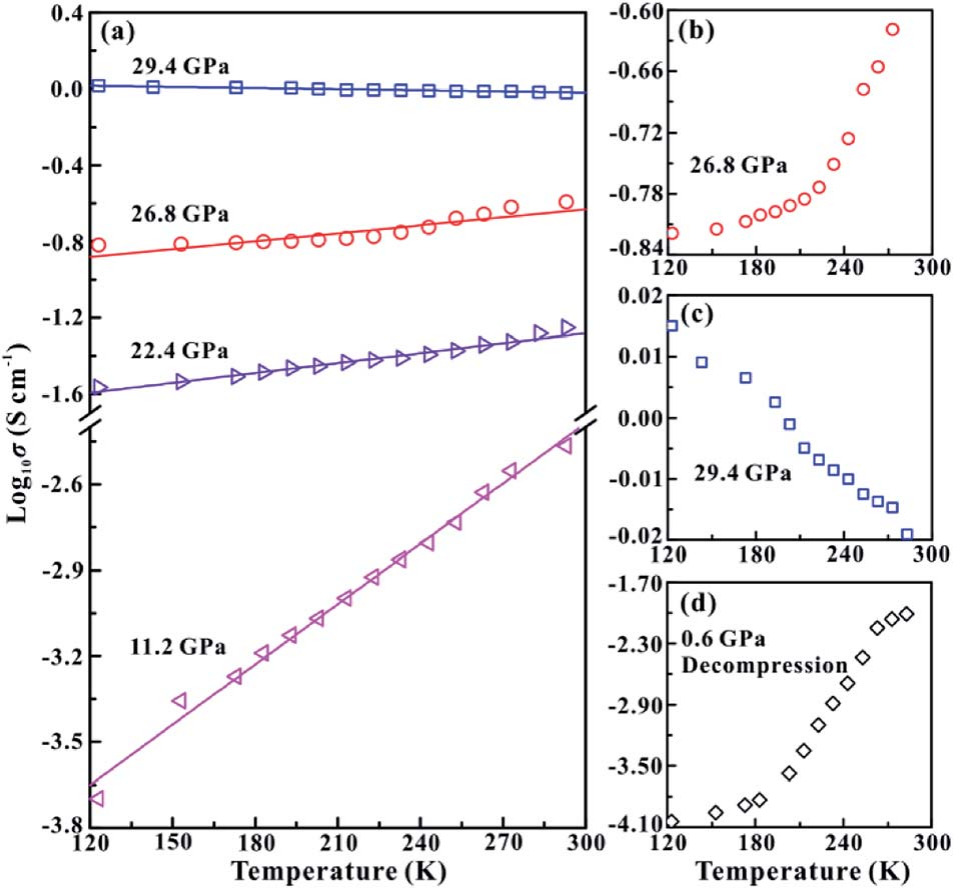
(a) The temperature dependence of electrical conductivity for MoSe_2_ at different pressure points under the hydrostatic condition. (b) The semiconducting state of MoSe_2_ at ∼26.8 GPa. (c) The metallization of MoSe_2_ at ∼29.4 GPa. (d) The semiconducting state of MoSe_2_ after decompression.

In order to further verify the metallization pressure point and examine whether MoSe_2_ endures an irreversible metallization, a series of temperature-dependent electrical conductivity measurements were carried out under the non-hydrostatic condition. Furthermore, we also conducted the temperature-dependent electrical conductivity measurements under the hydrostatic condition for comparisons. Generally, the semiconductor exhibits a positive relationship between electrical conductivity and temperature, while the metal does the opposite.^25,26^ Under the non-hydrostatic condition, the temperature dependence of electrical conductivity at various pressure points is shown in Fig. 4a. In the pressure range below ∼26.1 GPa, the electrical conductivity of MoSe_2_ increases with the increase in temperature, displaying a typical semiconductor characterization (Fig. 4b). By contrast, the electrical conductivity decreases with the increase in temperature above ∼26.1 GPa, indicating the occurrence of metallization at this point (Fig. 4c). Furthermore, it can be clearly observed from Fig. 4d that the sample still retains the metallic state after decompression, which further confirmed the irreversible metallization property for MoSe_2_ under these conditions. However, some different metallization characteristics of MoSe_2_ are obtained under the hydrostatic condition. As shown in Fig. 5a–c, the metallization pressure point is determined to be ∼29.4 GPa, which is higher than that observed under the non-hydrostatic condition, and the delay of metallization under the hydrostatic condition is in agreement with the above-mentioned FWHM results. Contrary to the irreversible metallization under the non-hydrostatic condition, MoSe_2_ undergoes a reversible metallization upon decompression under the hydrostatic condition (Fig. 5d). In a similar study, Zhao *et al.* observed the metallization phenomenon using the cubic boron nitride as the pressure medium for MoSe_2_ at a higher pressure of ∼40 GPa.^7^ It is possibly related to different experimental methods and pressure media, which can result in a discrepancy of metallization pressure points reported by us and Zhao *et al.*

Our electrical conductivity experimental results clearly indicate that the metallization behaviour of MoSe_2_ exhibits significant dependence on the pressure environments: under the hydrostatic condition, the metallization of MoSe_2_ occurs at ∼29.4 GPa and is reversible, while that under the non-hydrostatic condition occurs at a relatively lower pressure of ∼26.1 GPa and is irreversible. The key to understand these discrepancies in the metallization phenomenon is to figure out the states of the deviatoric stress and interlayer interaction under different hydrostatic environments, which have been proved to be very critical factors in influencing the metallization process of other similar layered materials.^13,14^ For the hydrostatic condition, the use of helium as the pressure-transmitting medium can alleviate the deviatoric stress and interlayer interaction through the injection of this medium into the interlayer space under high pressure. As a result, the delay of metallization occurs in the process of compression, and the interlayer distance recovers with the escape of pressure medium molecules from the layers after decompression. By contrast, strong deviatoric stress and interlayer interactions are generated within the vdW gap under the non-hydrostatic condition, which facilitates the occurrence of metallization and gives rise to the permanently plastic deformation of the interlayer spacing and thus the irreversible metallization.

**Fig. 6:**
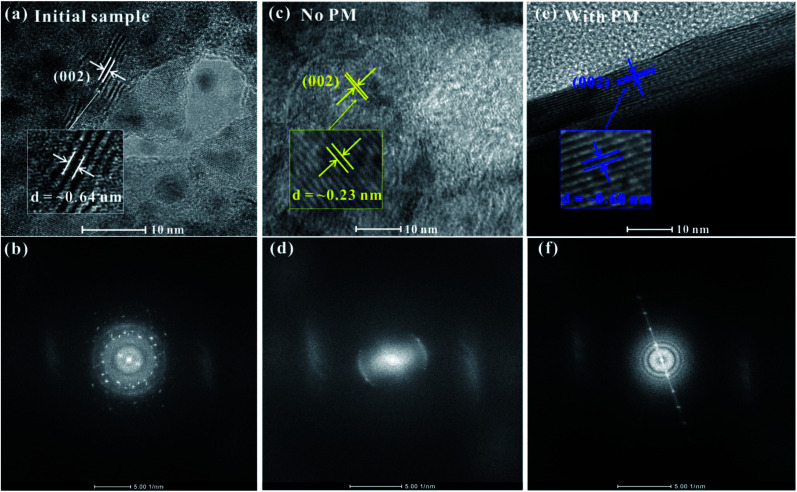
(a), (c) and (e) The HRTEM image of the starting sample, the recovered MoSe_2_ after decompression from ∼40.0 GPa under the non-hydrostatic condition, and the recovered MoSe_2_ after decompression from ∼39.6 GPa under the hydrostatic condition, respectively. (b), (d) and (f) The corresponding fast Fourier transform (FFT) pattern. Scale bar, 10 nm.

**Fig. 7:**
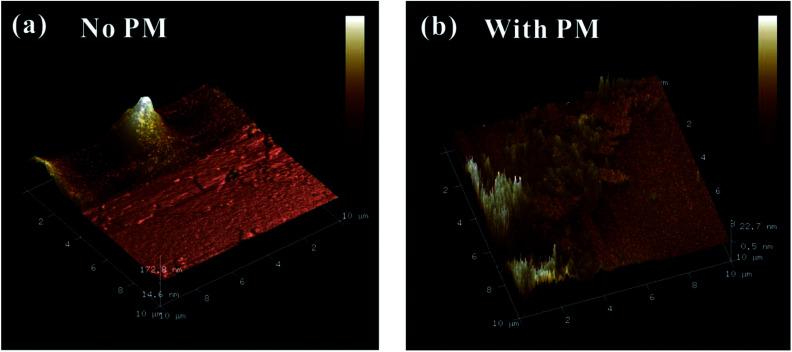
(a) AFM images of decompressed MoSe_2_ from ∼37.5 GPa under the non-hydrostatic condition. (b) AFM images of decompressed MoSe_2_ from ∼38.8 GPa under the hydrostatic condition.

To further check the pressure-induced metallization reversibility of MoSe_2_, we employed HRTEM and AFM to reveal the changes in the structure and morphology of recovered samples after decompression. From the HRTEM presented in Fig. 6a, the observed layers of the initial sample show a ∼0.64 nm interlayer spacing, which corresponds to the (002) oriented crystal planes of MoSe_2_ and is in good agreement with previous results.^27,28^ In the meantime, the corresponding fast Fourier transform (FFT) pattern is plotted in Fig. 6b, indicating the highly crystalline structure of the initial sample. After decompression from ∼40.0 GPa under the non-hydrostatic condition, the interlayer spacing of the sample drastically decreases to ∼0.23 nm (Fig. 6c). The corresponding FFT pattern shows a relatively weak halo ring of the (002) crystal planes, implying a low-degree crystalline structure for MoSe_2_ after the non-hydrostatic compression (Fig. 6d). However, under the hydrostatic condition, the recovered sample exhibits a well-discernible layered structure with an interlayer spacing of ∼0.60 nm, and the FFT pattern reveals a well-preserved crystal structure for the recovered MoSe_2_ after the hydrostatic compression (Fig. 6e and f). This indicated that the change in the interlayer spacing is irrevocable under the non-hydrostatic conditions; as a result, the irreversible metallization is observed. However, owing to the introduction of the pressure medium molecules in the interlayer spacing, the interlayer interactions and deviatoric stress are alleviated, causing the change in interlayer spacing to be recoverable under the hydrostatic condition and thus the reversible metallization of MoSe_2_. Meanwhile, according to the analysis of AFM, the surface morphology of recovered samples shows some significant differences under non-hydrostatic and hydrostatic conditions (Fig. 7a and b). After decompression from ∼37.5 GPa under the non-hydrostatic condition, the surface morphology of MoSe_2_ was very lumpy and the layered structure was destroyed. In contrast, for the recovered sample under the hydrostatic condition, the layered structure of MoSe_2_ could be well preserved as a result of the protection of the pressure medium. All of the HRTEM and AFM results provide favourable evidences for the irreversible metallization under the non-hydrostatic condition and reversible metallization under the hydrostatic condition.

**Fig. 8:**
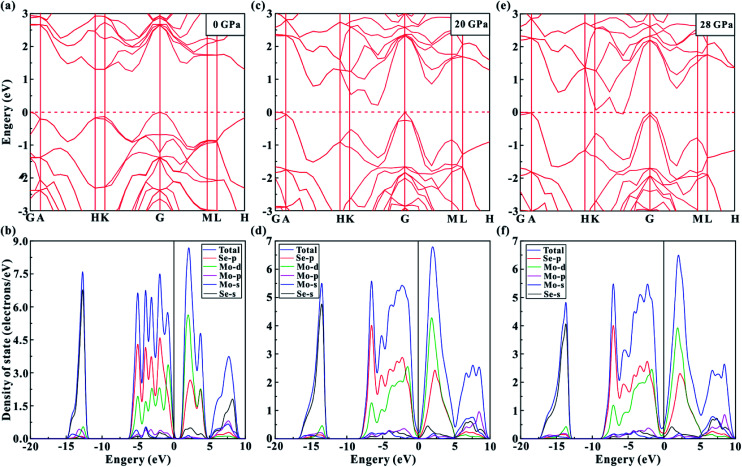
(a), (c) and (e) The calculated band structure of MoSe_2_ at representative pressures of ∼0, ∼20 and ∼28 GPa, respectively. (b), (d) and (f) The corresponding total density and projected density at ∼0, ∼20 and ∼28 GPa, respectively.

**Fig. 9:**
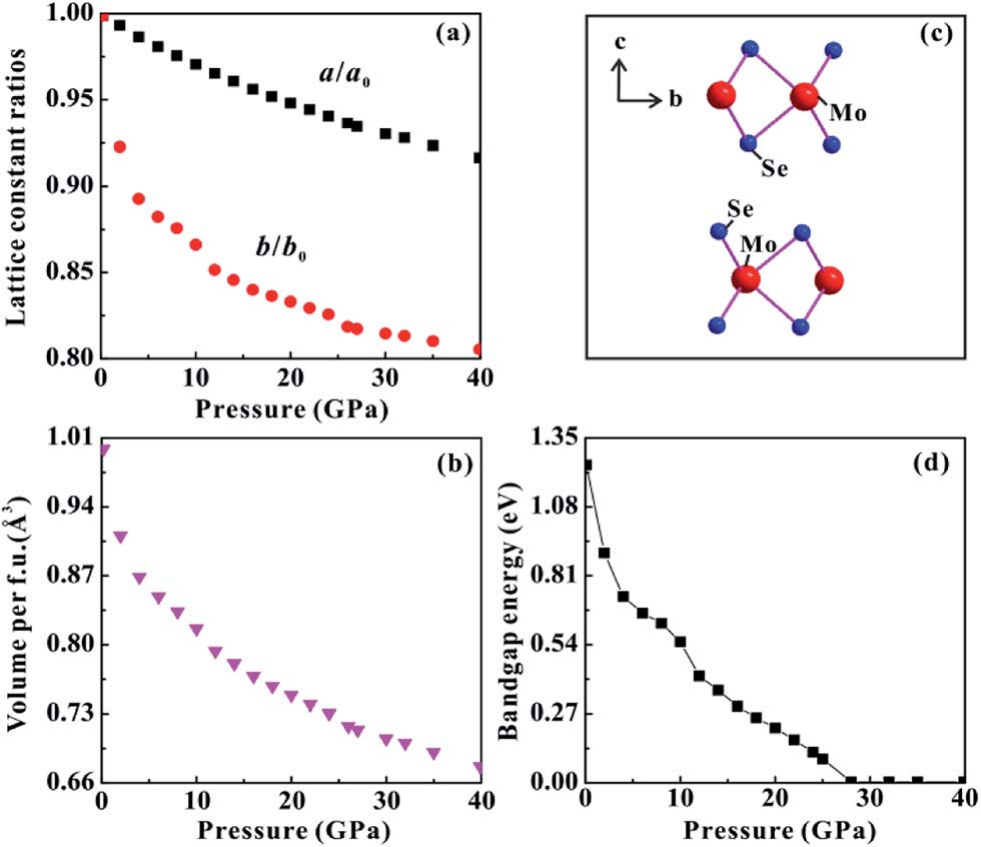
(a) The pressure-dependent *a*/*a*_0_ and *c*/*c*_0_ data of MoSe_2_ from theoretical calculations. (b) The calculated volume of MoSe_2_*versus* pressure. (c) Crystalline structure of hexagonal (*P*6_3_/*mmc*) crystal MoSe_2_ at ambient pressure. (d) The calculated bandgap of MoSe_2_ with increasing pressure up to 40.0 GPa.

The theoretical calculations were implemented to predict the electronic and structural evolutions of MoSe_2_ under high pressure. Fig. 8 presents the predicted band structure, total density and projected density of MoSe_2_ at three representative pressure points (0, 10 and 28 GPa). As shown in Fig. 8a, the calculated band structure results clearly reveal a semiconducting ground state for MoSe_2_ with a bandgap of 1.24 eV at atmospheric pressure, which is slightly higher by ∼0.2 eV than the previously reported result.^29^ According to first-principles theoretical calculations by Ruiz-Fuertes *et al.*, the feeble discrepancy is tolerant.^30^ The corresponding density of state for MoSe_2_ at atmospheric pressure is plotted in Fig. 8b. The valence bands between about −15 and −12 eV are predominantly composed of the Se-s state. From about −6 to 0 eV, the valence bands are composed of Se-s, Se-p, Mo-s, Mo-p, and Mo-d states, which are mutually hybridized and dominated by the Se-p and Mo-d states. Above the Fermi-level (*E*_F_), the lowest conduction bands from about 0 to 5 eV are dominated by the Mo-d and Se-p states, and the highest conduction bands from about 5 to 10 eV are mainly composed of the Se-s state. When the pressure is enhanced to 20 GPa, the bandgap of MoSe_2_ decreases to 0.22 eV (Fig. 8c). Meanwhile, all of these energy bands have been expanded at this pressure point, and the high-energy valence bands widen more than the conduction bands (Fig. 8d). Upon further compression to 28 GPa, the bandgap reaches a critical value of 0 eV, and an overlap between valence and conduction bands can be clearly observed (Fig. 8e and f), showing the metal characteristic for MoSe_2_. Fig. 9d displays the predicted pressure dependence of the bandgap up to 40 GPa. The bandgap energy of MoSe_2_ shows a decreasing tendency with the increasing pressure and reaches 0 eV at a pressure of 28 GPa, an indication of metallization for MoSe_2_ at this pressure point. This predicted metallization pressure point by the generalized gradient approximation (GGA) is well consistent with our above-mentioned experimental results. However, its magnitude is obviously lower than that reported by Zhao *et al.*, which was obtained by the HSE06 hybrid functional approximation.^7^ As usual, the bandgap energy might be overestimated by HSE06 hybrid functional approximation.^10^ In addition, the calculated pressure-dependent normalized cell parameters and volume of MoSe_2_ are presented in Fig. 9a and b. It can be clearly seen that all of these obtained crystalline parameters including *a*/*a*_0_, *b*/*b*_0_ and unit cell volume for MoSe_2_ gradually decrease with the increase in the pressure. Obviously, all available continuous variations between crystalline parameters and pressure realized that the occurrence of the pressure-induced metallization for MoSe_2_ is not triggered by the structural phase transition. We think that the metallization phenomenon of MoSe_2_ is closely associated with the pressure-induced reduction of the intralayer distance, *i.e.*, with the increasing pressure, the distance of the neighbouring Se atom planes will be rapidly decreased, which results in the strong Se–Se interactions and further leads to the occurrence of metallization. In addition, a series of representative selenides with similar layered structures (*e.g.*, InSe, GaSe, ReSe_2_ and SnSe_2_) have also been reported to undergo the phase transition of metallization under high pressure, and therefore, the pressure-induced metallization maybe widely existing in most of selenides.^31–34^

## Conclusions

The high-pressure vibrational and electronic properties of MoSe_2_ were systematically investigated under non-hydrostatic and hydrostatic conditions using a diamond anvil cell in conjunction with Raman spectroscopy, electrical conductivity, atomic force microscopy, high-resolution transmission electron microscopy, and first-principles calculations. The results confirmed the electronic transition of MoSe_2_ from a semiconductor to a metal on the basis of the obvious changes in the FWHM of Raman modes, electrical conductivity and calculated bandgap energy. The metallization pressure points under non-hydrostatic and hydrostatic conditions were well determined to be ∼26.1 and ∼29.4 GPa, respectively. Interestingly, the metallization was irreversible under the non-hydrostatic condition, while that was reversible under the hydrostatic condition. The reversibility of metallization displayed by MoSe_2_ under different hydrostatic environments was attributed to the effect of pressure medium molecules, which played a critical role in determining whether the interlayer spacing can be restored upon decompression.

## Conflicts of interest

There are no conflicts to declare.

## Supplementary Material

RA-009-C8RA09441A-s001
