# Supplementary material for: Pressure-induced metallization in MoSe2 under different pressure conditions
Source: RSC Adv. 2019 Feb 15;9(10):5794–803. doi: 10.1039/c8ra09441a (PMC9060785; doi:10.1039/c8ra09441a)
Supplement: RA-009-C8RA09441A-s001 [file RA-009-C8RA09441A-s001.pdf]

Electronic Supplementary Material (ESI) for RSC Advances.
This journal is © The Royal Society of Chemistry 2019

Supplementary Information

Pressure-induced metallization in $MoSe_2$ under different pressure conditions

Linfei Yang,[ab] Lidong Dai,*[a] Heping Li,[a] Haiying Hu,[a] Kaixiang Liu,[ab] Chang Pu,[ab] Meiling Hong[ab] and Pengfei Liu[c]

[a] *Key Laboratory of High-Temperature and High-Pressure Study of the Earth's Interior, Institute of Geochemistry, Chinese Academy of Sciences, Guiyang, Guizhou 550081, China*

[b] *University of Chinese Academy of Sciences, Beijing 100049, China*

[c] *State Key Laboratory of Structural Chemistry, Fujian Institute of Research on the Structure of Matter, Chinese Academy of Sciences, Fuzhou, Fujian 350002, China*

*Corresponding author. *Email address*: dailidong@vip.gyig.ac.cn.

Supplementary Figures

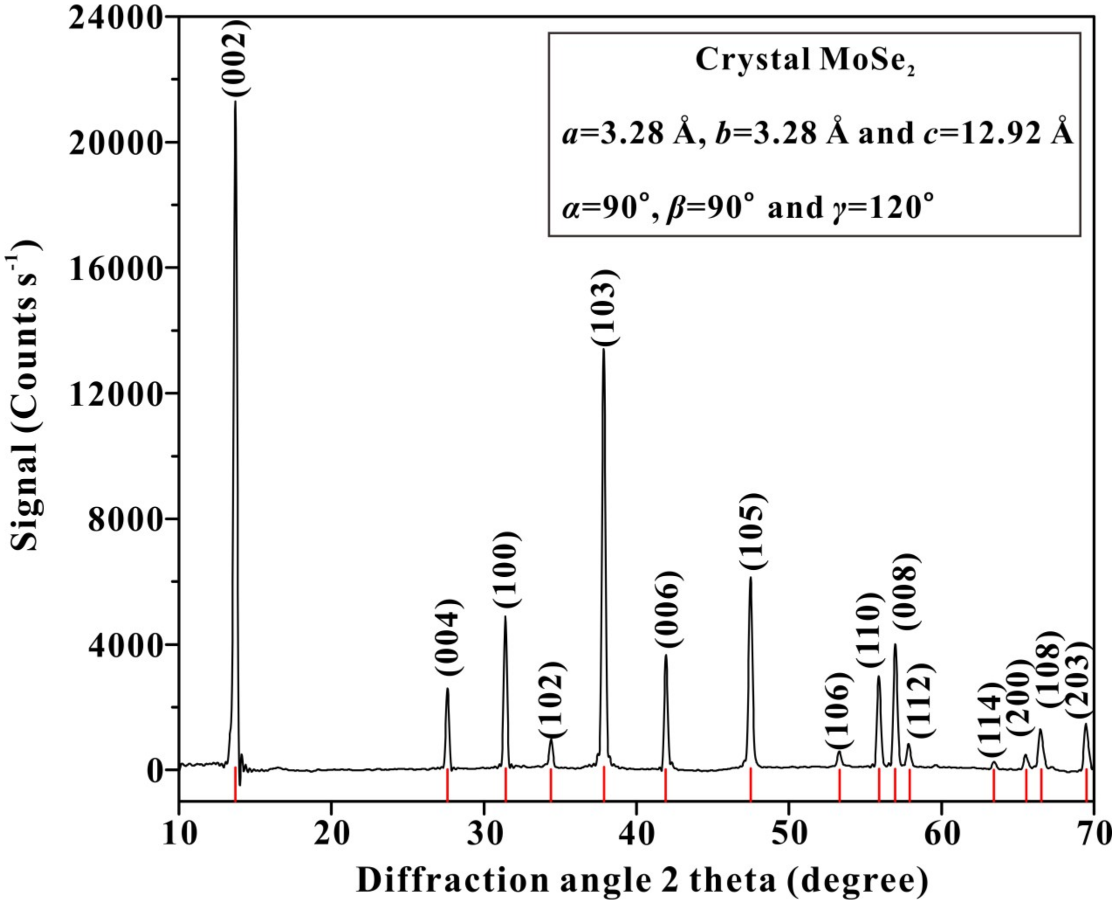

Fig. S1 The X-ray powder diffraction of crystal $MoSe_2$ at ambient conditions.

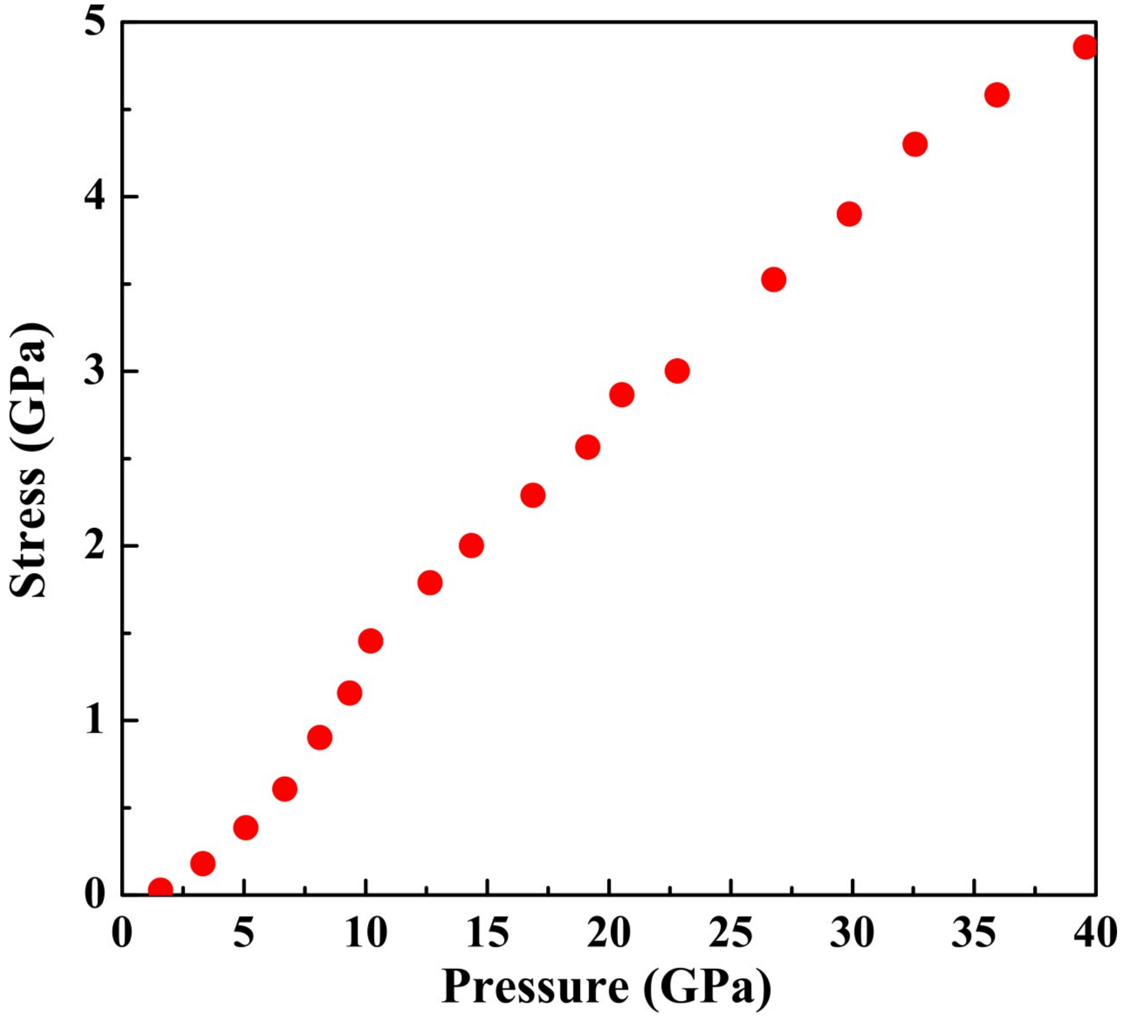

Fig. S2 The uniaxial stress of sample at the pressure range of 1.5–39.6 GPa.
